# Exploring *in-vivo* infrared spectroscopy for nail-based diabetes screening

**DOI:** 10.1364/BOE.520102

**Published:** 2024-02-28

**Authors:** Daniela Lazaro-Pacheco, Philip F Taday, Päivi Maria Paldánius

**Affiliations:** 1 University of Exeter, Engineering Department, Harrison Building, North Park Rd, Exeter EX44QF, United Kingdom; 2 Glyconics Limited, The Grosvenor, Basing View, Basingstoke RG214HG, United Kingdom; 3 Children’s Hospital, University of Helsinki and Helsinki University Hospital, Helsinki, Finland; 4 Research Program for Clinical and Molecular Metabolism, University of Helsinki, Helsinki, Finland

## Abstract

Diabetes screening is traditionally complex, inefficient, and reliant on invasive sampling. This study evaluates near-infrared spectroscopy for non-invasive detection of glycated keratin in nails in vivo. Glycation of keratin, prevalent in tissues like nails and skin, is a key indicator of T2DM risk. In this study involving 200 participants (100 with diabetes, 100 without), NIR’s efficacy was compared against a point-of-care HbA1c analyzer. Results showed a specificity of 92.9% in diabetes risk assessment. This study highlights the proposed NIR system potential as a simple, reliable tool for early diabetes screening and risk management in various healthcare settings.

## 1. Introduction

The global burden of diabetes mellitus (DM) presents significant challenges to healthcare systems worldwide. In 2021, healthcare costs attributed to diabetes soared to USD 966 billion, marking a staggering 316% rise in the past 15 years [1]. The most frequent type of DM, type 2 diabetes (T2DM), is characterized by chronic hyperglycemia and insulin resistance, leading to chronic complications adversely affecting the quality of life and life expectancy of the affected individuals [2]. Effective and impactful strategies for early detection, prevention, diagnosis, and management of T2DM are fundamental, as they yield substantial benefits for global health equity and development. Early detection and monitoring of risk of T2DM are crucial for effective management and prevention, or at least slowing down of its progressive complications. However, the current diagnostic methods, primarily reliant on blood-based assays, pose challenges regarding accessibility, cost, and even accuracy beyond the limited populations in which they were validated and healthcare access inequality.

Routine screening is advocated for identifying individuals at risk of T2DM due to the availability of established diagnostic methods. Early detection can lead to early intervention and even prevention of the condition through lifestyle modifications, dietary intervention and even medication, which can significantly slow down the disease's progression and reduce its complications [3]. T2DM can be a silent disease and progress slowly over decades while the affected individual experiences no or limited symptoms of even sustained hyperglycemia. The International Diabetes Federation (IDF) estimates indicate that approximately 541 million individuals are living with undiagnosed impaired glucose tolerance (IGT), a pre-diabetes stage in which fasting glucose values are still within normal range and thus masking the progressively developing condition if only conventional methods for detection are implemented [1]. There is evidence that lifestyle and drug interventions [4,5] in people with IGT and impaired fasting glucose (IFG) can postpone the onset of T2DM [6]. Additionally, early detection and intervention have been demonstrated to lead to slower progression of the underlying disease and return of glycemic levels closer to normal values [7]. However, screening for T2DM may not directly lower mortality rates over a decade of follow-up [3,8]; however, this evidence was based on the accuracy of the conventional methods in a primarily White Caucasian (male) population only. The face of diabetes is changing, and we must be able to detect the risk of underdiagnosed or early diabetes in our increasingly diverse populations at risk.

Currently, wide-scale screening for diabetes in diverse populations with unknown or unpredictable risk for diabetes is not cost-efficient due to the polyfactorial manifestation of diabetes in individuals without apparent risk factors. However, due to the estimated high prevalence of underdiagnosed diabetes especially among people with limited access to public healthcare infrastructure and socioeconomic inequity, more affordable, accessible, and sustainable screening methods must be developed to curb the diabetes epidemic among these communities. Diabetes affects people of color at higher rates than White Caucasians [2], and providers of healthcare need to be versed in addressing social determinants of health as well as cultural and societal competencies.

In 2021, IDF estimated that one in ten adults worldwide were living with diabetes while for every diagnosed individual, there are potentially one to three others with undiagnosed diabetes. This prevalence of underdiagnosis was highlighted in a real-world setting with a study which randomly screened over 1,300 presenting at A&E, irrespective of their presenting condition. The study found that 9% had sustained hyperglycemia, indicative of previously undiagnosed diabetes, while about 30% were identified living with pre-diabetes [9]. These screening opportunities could make an important contribution to identifying undiagnosed individuals who will benefit from early diabetes treatment and lifestyle changes and thus reduce their risks of long-term complications.

Under sustained hyperglycemic conditions, the accumulation of advanced glycation end-products (AGEs) has been demonstrated in various tissues [10–14]. High levels of AGEs in diabetes have been attributed to endogenous sources such as hyperglycemia and tissue damage caused by reactive oxygen radicals [15]. AGEs, reflecting long-term glycemic control, can be effectively detected through spectroscopic techniques. Infrared spectroscopy emerges as a promising alternative for diabetes screening through the non-invasive glycation assessment of tissues [16,17]. This technique, which analyses molecular vibrations that correlate to specific chemical bonds, offers a rapid and patient-friendly, non-invasive approach. The integration of label-free, highly specific techniques into diverse sectors such as food analysis [18], pharmaceutical quality control [19], and environmental monitoring [20] has proven successful in the past. These methods are now gradually making inroads into the medical field, with applications ranging from biomarker detection to pathological diagnostics.

Near-Infrared (NIR) spectroscopy offers a unique lens through which the composite biomolecular makeup of tissues can be examined. The technique yields spectra that elucidate the aggregate composition of biomolecules, underscoring the intricate and varied nature of tissue constituents. This inherent complexity and heterogeneity pose significant challenges in segregating signals attributable to individual molecules from within a conglomerate sample. Despite this challenge, the field has developed a robust understanding of the spectral assignments associated with broad categories of biomolecules, such as carbohydrates, proteins, nucleic acids, and lipids [21–23]. These assignments give us a valuable indication of the general biomolecular composition of the tissues we study, even if they do not allow for the identification of specific single molecules. The multifaceted influences on spectral characteristics, stemming from the biological matrix's complexity, underscore the need for a detailed and discerning analysis of spectral data to accurately interpret the biochemical underpinnings of observed phenomena. Incorporating multivariate analysis and chemometric techniques enhances our ability to explore potential areas of interest and identify spectral correlations within large datasets, thereby enriching our interpretation of the biochemical dynamics at play.

The dynamic nature of fingernail plates and their continuous growth, typically at a rate of 3.5 mm per month, presents an intriguing avenue for diagnostic investigation. The complete natural regeneration of a fingernail, which occurs over a period of 4 to 6 months following avulsion [24], provides a temporal window into the physiological state of the body. Fingernails, composed of up to 85% of keratin proteins, are susceptible to glycation, which may reflect average blood glucose levels over several months [17]. Nails, as a keratin-rich tissue, accumulate AGEs over time, providing a unique matrix for assessing long-term glycemic accumulation. Using nails for glycation assessment via NIR spectroscopy presents a novel approach to diabetes screening as it is underpinned by the ease with which they can be non-invasively assessed, their stability, and the minimal requirements for measurement preparation. However, this method faces challenges, including the variability in geometry and the influence of external factors such as contaminants on spectroscopic readings. A critical consideration in nail assessment is the possibility of long-term biochemical alterations in the nails due to the repeated use of cosmetic products like nail varnish. However, research into the impact of such cosmetic treatments on fingernail plates has shown that applying nail varnish does not significantly change the nail's chemical composition once the varnish is removed. This conclusion is supported by the uniformity of carbon-13 spectral data between nails that have been treated with varnish and those that have not, indicating that the underlying chemical structure of the nail plate remains unchanged post-treatment [24]. Despite these challenges, the potential for nails as a non-invasive biomarker for glycemic control remains a promising avenue for exploration.

Prior research has established a foundation for distinguishing specific spectroscopic signatures characteristic of AGEs, detectable in particular vibrational bands. The glycation process is associated with spectral shifts within the 4300 to 4400 cm^−1^, 5270 cm^−1^, and between 6600 and 7500 cm^−1^ regions [17]. Additionally, studies have demonstrated that individuals with diabetes, irrespective of their disease management status, display an elevated concentration of certain AGE products, such as carboxymethyl-lysine (CML) in their nails, as determined through infrared spectroscopy. This analysis particularly emphasizes the spectral positions of Amide I, Amide II, and disulfide linkages within the keratin structure of the fingernail plate [25].

A comparative examination of spectroscopic data across varying degrees of nail tissue glycation has been instrumental in pinpointing spectral regions of interest (ROI) that manifest glycation-induced alterations [17,26]. For example, spectral modifications, including peak widening around the 4300-4400 cm^−1^ area, are correlated with heightened glycation levels, indicating that these spectral shifts may act as markers for the presence of higher glycated proteins within the nail. The accumulated evidence supports the utility of spectroscopic techniques in the investigation and potential non-invasive screening for conditions associated with increased AGE accumulation, such as diabetes, especially when integrated with sophisticated predictive methodologies.

This study explores the potential of NIR spectroscopy in providing detailed chemical insights associated with glycated products in nails related to the presence of hyperglycemia and diabetes risk.

## 2. Methods

In this study, a hand-held near-infrared diffuse reflectance (NIR) miniaturized spectrometer, SYS-IR-R-P, (trinamiX, Germany), featuring a sapphire glass specimen interface with infrared collection optics, and a removable reference standard reflector for daily calibration. The unit connects via Bluetooth to a mobile device for data transfer and was used to obtain spectral data from participant’s nails. The spectrometer uses 6 halogen lamps in a circulator arrangement placed around a linear lead sulfide (PbS) sensor. The PbS sensor comprises a 256-pixel array on which is placed a linear variable filter (LVF). This has been designed to cover a wavelength range of 1400 nm to 2500 nm. Each pixel integrates responses over a wavelength region of 5 nm. A Zenith polymer wavelength standard (SG3334, Sphereoptics, Germany) is used to calibrate the wavelength and to confirm the position of the LVF in front of the sensor. For each measurement, three individual measurements are undertaken: the dark signal Sd (l), the white signal from a 100% target, Sw (l), and the sample spectrum, S(l). The reflectance spectrum is then given by

(1) $$R(l)=\frac{S(l)-\;S_{d}(l)}{S_{w}(l)-\;S_{d}(l)}$$

The NIR device is equipped with advanced quality control algorithms to guarantee precise calibration. The algorithms ensure accurate measurements by prompting re-calibration via the mobile user interface if internal checks detect failures, maintaining the device's calibration within required specifications. Additionally, the device incorporates a thermoelectric cooler, utilized by its PbS detector, to maintain a consistent temperature of 15°C. This feature is critical for achieving precise temperature stabilization, with residual temperature fluctuations kept below 0.015°C.

**Fig. 1.**
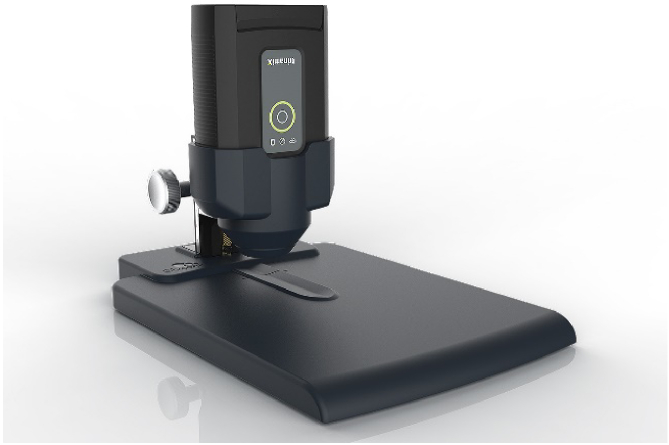
NIR system for nail assessment comprising of positioning cradle and NIR spectrometer.

The sensor head, weighing approximately 500 grams, can be cumbersome for extended use. To mitigate this, a custom-made, interoperable cradle was designed to optimally position the spectrometer during data acquisition. This setup not only ensures precise alignment and consistent signal reflection (Fig. 1), but also maintains clear visibility of the target area (center of the nail), enhancing the overall ease and safety of use.

**Fig. 2.**
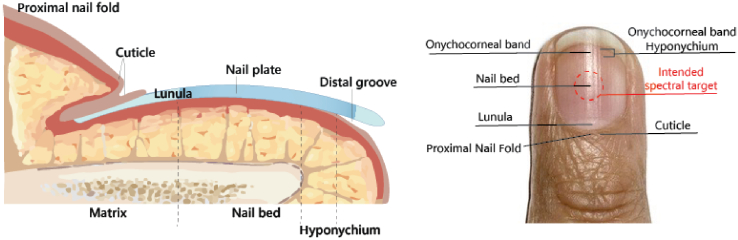
Nail structure.

Human fingernails are primarily composed of keratin, and contain small quantities of lipids (0.1–1.0 wt%, predominantly cholesterol), minerals (with chlorine, calcium, potassium, sodium, silicon, magnesium, zinc, iron, aluminum, bromine, and copper being the most abundant), and water (constituting 7–12 wt%) [24]. The nail unit is composed of the proximal nail fold, cuticle, lunula (the white crescent-shaped area at the base of the nail), nail plate, distal groove, matrix (the tissue under the nail that produces cells that become the nail plate), nail bed, and hyponychium (the area beneath the free edge of the nail) as shown in Fig. 2. Focusing and targeting the center of the nail when assessing using NIR spectroscopy is recommended because the nail plate there is typically more uniform in thickness and composition. This uniformity minimizes variability that can arise from edges like the lunula or the distal groove, which may have different properties and could potentially influence the NIR readings.

Assessing the center ensures a more consistent and representative sample of the nail's overall condition. To ensure the data acquisition is centered on the nail, the cradle features a holder for the spectrometer equipped with a targeting aperture (10 mm). This not only provides visual confirmation of the specific area for measurement but also, due to its design, accommodates various finger shapes by allowing clearance above the proximal fold. This effectively isolates the nail from the cuticle and surrounding skin, ensuring that measurements are exclusive to the nail plate. Furthermore, the base of the cradle incorporates two intersecting grooves that guide finger placement, marking the central targeting area along the XY axes for precise data acquisition.

Nail plates predominantly comprise hard α-keratins, accounting for 80–90% of their structure, with the remainder comprising soft α-keratins [24]. Given this composition, the spectral assessment was carried out on the 4080-6896 cm^−1^ (2.450-1.450 µm) spectral range. With particular focus on the 1.5-2.2 µm and 2.2-2.5 µm spectral ranges. Comparatively, the 1.5-2.2 µm region may offer general information about bond types and basic molecular structures, whereas the 2.2-2.5 µm region could provide more specific insights into molecular alterations due to glycation.

■ **1.5 -2.2 µm range (4545-6667 cm^−1^)**: This segment of the NIR spectrum, known for its sensitivity to O-H, N-H, and C-H bond vibrations, aligns with the first overtone and combination bands of molecular vibrations. It is highly relevant for examining glycated proteins, providing insights into hydrogen-bonding environments and the presence of functional groups linked to protein structures and modifications. Absorption patterns in this region could indicate glycation levels in proteins.
■ **2.2 - 2.5 µm range(4000-4545 cm^−1^)**: Closer to the mid-infrared spectrum, this region showcases stronger absorption features, especially sensitive to heavier atom vibrations like C = O and C-N, common in proteins. This segment could reveal details about advanced glycation end-products (AGEs) accumulation and protein conformation changes, as absorption here is influenced by alterations in protein conformation and the environment around glycation sites.

Participants included in the clinical investigation were apparently healthy adults aged 18 years or older, either with known or unknown T2DM status (50:50). They were required to provide written consent and undergo glycaemia assessment through both Near-Infrared (NIR) spectra assessment and HbA1c finger prick test (analyzed with a point-of-care HbA1c analyzer, QuoTest). A critical requirement for inclusion was the possession of two healthy, undamaged, and intact fingernails on both the right and left hand middle fingers, as visually assessed.

Individuals were excluded from the study if they had any known medical conditions that could impact the assessment methodology for glycaemia. These conditions included anemia (iron deficiency, sickle cell anemia, etc.), haemoglobinopathies or atypical hemoglobin subtypes not detectable by regular assays, severe renal impairment (Chronic Kidney Disease stages III-IV), decompensated hepatic disease, severe Vitamin D deficiency, known severe immunodeficiency such as HIV, malignancy, or other chronic conditions significantly impacting glycaemia assessment. Participants with eating disorders (as per clinical assessment), those who had donated blood within the last 28 days, or individuals with any other type of diabetes (including Type 1 Diabetes Mellitus) were also excluded.

Further exclusion criteria included structural, deviating, and visually detectable deviations in the appearance of the nails potentially impacting the spectra measurement. This included nail dystrophy or deformity, severe nail infections (onychomycosis causing visual changes in the appearance of the nail), rare hereditable conditions impacting the structure of keratin, mechanical damage or marks on the surface of the nail after removal of nail polish, and the use of acrylic or gel nail decoration and polish that could not be removed. Any systemic or topical medication known to modify either the surface or structure of the nail or the accuracy of the HbA1c value also led to exclusion from the study.

These pilot studies for this research commenced after receipt of applicable ethical and regulatory approvals (ANODE01: NCT05198895 and ANODE02:NCT05476016) at two distinct locations: ANODE01 at the Leicester Diabetes Centre in the United Kingdom, and ANODE02 at the Sant Martí Primary Health Center in Barcelona, Spain. Each pilot study enrolled 100 participants (total n = 200 for two studies) with half of the participants with a known T2DM status.

**Fig. 3.**
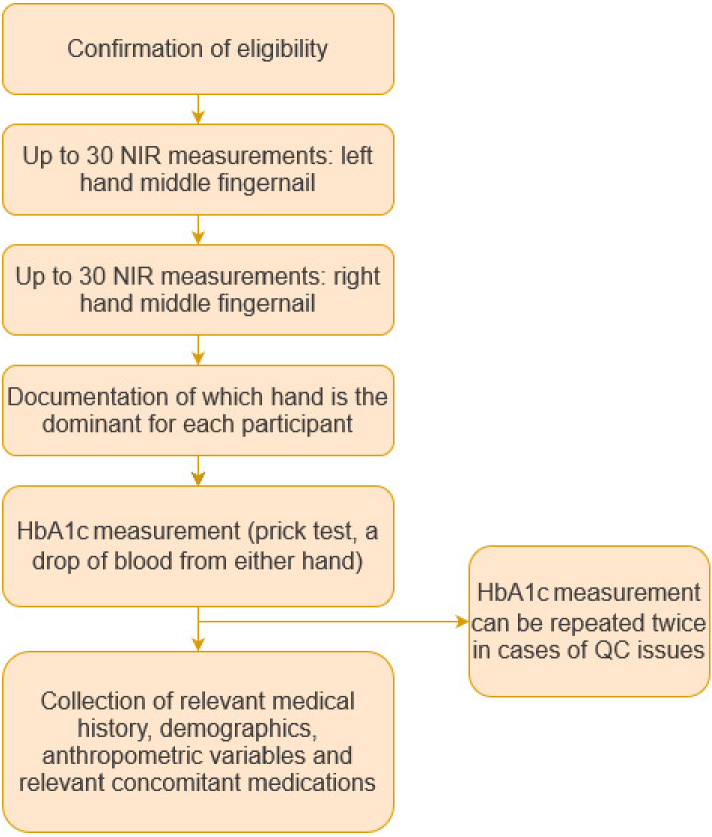
Pilot study methodology for data collection.

After confirming eligibility and visual assessment of fingernails, participants underwent up to thirty NIR spectra measurements per middle fingernail on both hands, totaling up to 60 measurements each (Fig. 3). During measurements, participants were seated, placing their finger on the cradle as instructed. Unique participant identifiers in the form of pre-assigned QR codes ensured accurate recording and labeling of each measurement.

Following the NIR spectra measurements, HbA1c levels were assessed using the Quo-Test device (certified to NGSP and IFCC standards) with single-use test cartridges based on the manufacturer's instructions. This test was performed on any finger and hand, and could be repeated up to two times in case of technical issues.

Dichotomized diabetes risk status, defined as HbA1c levels less than 48 mmol/mol (below 6.5%), was assessed as primary endpoint. Additionally other cut-off values indicative of normal or elevated values within pre-diabetic range were being assessed. These thresholds were selected based on the diagnostic criteria established by the American Diabetes Association [27] and most international diabetes associations as shown in Table 1. The assessment employed chemometric prediction models to evaluate the relationship between clinical specificity and sensitivity alongside true and false positive outcomes. The chemometric modelling involved up to six predictive models employing Partial Least Squares Discriminant Analysis (PLS-DA). These models were trained with a dataset distribution of 80% for training and 20% for validation to ascertain their robustness and insensitivity to baseline patient characteristics. Validation of these models was executed through 7-fold cross-validation.

**Table 1.** Criteria for pre-diabetes according to the American Diabetes Association (ADA) [27]

Definition	HbA1c value
Normal	< 5.7% / 39 mmol/mol
Pre-diabetes	5.7–6.4% / 39- 47 mmol/mol
Diabetes	≥ 6.5% / 48 mmol/mol

In evaluating the optimization of chemometric algorithms, the focus was on improving sensitivity and specificity, with a greater emphasis on specificity due to the system's intended use for screening rather than diagnosis. This prioritization guided the selection of the most suitable model. The categorizing process was based on the majority of each participant's predictions into one of two classes: those with HbA1c levels lower than 6.5% and those with levels equal to or above 6.5%. As individuals with established T2DM were mostly well-controlled and their glycemic values were below diagnostic levels, multiple sensitivity analyses based on glycaemia rather than T2DM status could be performed. A singular status per participant was then employed to compute sensitivity and specificity, thereby evaluating the overall performance of the classification system.

**Fig. 4.**
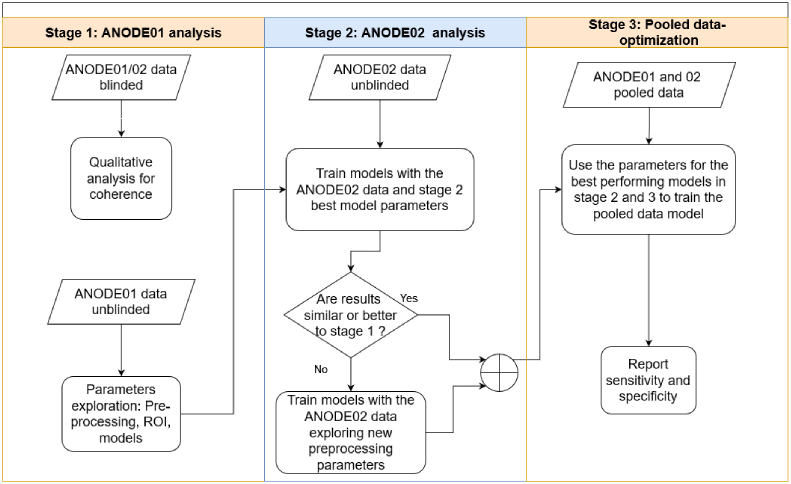
Staged data analysis process.

The optimization process, as illustrated in Fig. 4, unfolded in multiple stages. In the initial stage, a qualitative analysis of the data was conducted to ensure coherence across spectra within a hand and between both hands of the same participant. Concurrently, spectral data from ANODE01 and 02 was used to explore pre-processing parameters, regions of interest (ROI), and model calibration. Progressing to Stage 2, the parameters identified in Stage 1 were employed to train models using data from ANODE02. If performance was comparable or superior to the preceding stage, the process advanced to Stage 3. In cases where performance fell short, additional optimization steps were undertaken. In the final stage (Stage 3), data from both pilot studies was pooled and the optimal parameters from Stages 1 and 2 were assessed for model training, with subsequent evaluation of sensitivity and specificity to gauge overall performance.

The chemometric exploration and optimization were completed using SIMCA 17.0 (Sartorius, Sweden) followed by subsequent statistical analyses performed using MATLAB 2023b and SAS.

## 3. Results and discussion

The two pilot studies provided over 12,000 NIR spectral readings which were pooled for a pre-planned combined analysis. The participant demographics (Table 2) revealed a predominantly female cohort (58.5%), with a majority being non-smokers (80.0%). This diverse group had a median age of 58 years, ranging from 19 to 94 years. The median Body Mass Index (BMI) was 27.3 kg/m^2^, with a spread from 17 to 44. For participants with diabetes, the median HbA1c (%) was 7.0 (interquartile range: 6.3-7.8), while for those without diabetes, it was 5.4 (range: 5.2-5.6).

**Table 2.** Demographics and baseline characteristics

Parameter		Type II DM Status		Total (N = 200)
		DM (N = 100)	non-DM (N = 100)	
Age (years)	n	100	100	200
	Mean (SD)	67.4 (11.95)	46.9 (17.11)	57.2 (17.96)
	Median	68.5	47	58
	Q1 - Q3	61 - 75	32 - 58	45 - 72
	Minimum - Maximum	36 - 94	19 - 90	19 - 94
Sex [n (%)]	Female	44 (44.0)	73 (73.0)	117 (58.5)
	Male	56 (56.0)	27 (27.0)	83 (41.5)
Race [n (%)]	Asian or Ethnic Asian	15 (15.0)	19 (19.0)	34 (17.0)
	Black, African, Caribbean or Ethnic Black	2 (2.0)	2 (2.0)	4 (2.0)
	Caucasian	78 (78.0)	72 (72.0)	150 (75.0)
	Mixed or Multiple Ethnic Groups	2 (2.0)	3 (3.0)	5 (2.5)
	Other	3 (3.0)	4 (4.0)	7 (3.5)
Weight (kg)	n	100	98	198
	Mean (SD)	82.0 (16.88)	72.1 (15.86)	77.1 (17.08)
	Median	80	70	74
	Q1 - Q3	70.0 - 95.9	60.0 - 80.0	65.0 - 87.0
	Minimum - Maximum	51.0 - 128.8	47.0 - 130.0	47.0 - 130.0
Height (m)	n	100	99	199
	Mean (SD)	1.66 (0.095)	1.66 (0.097)	1.66 (0.095)
	Median	1.67	1.64	1.66
	Q1 - Q3	1.59 - 1.74	1.59 - 1.73	1.59 - 1.73
	Minimum - Maximum	1.46 - 1.85	1.45 - 1.92	1.45 - 1.92
BMI (kg/m2)	n	100	98	198
	Mean (SD)	29.63 (5.267)	26.16 (4.885)	27.91 (5.358)
	Median	29.08	25.35	27.34
	Q1 - Q3	25.19 - 32.76	22.86 - 28.89	23.95 - 31.34
	Minimum - Maximum	18.07 - 44.27	17.30 - 41.91	17.30 - 44.27
HbA1c (%)	n	100	99	199
	Mean (SD)	7.36 (1.619)	5.42 (0.345)	6.39 (1.523)
	Median	7	5.4	5.9
	Q1 - Q3	6.30 - 7.75	5.20 - 5.60	5.40 - 7.00
	Minimum - Maximum	5.30 - 16.90	4.60 - 6.70	4.60 - 16.90

**Fig. 5.**
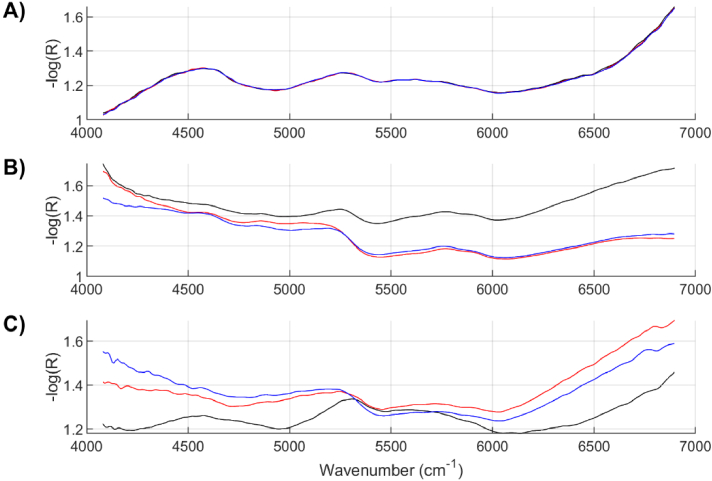
NIR spectra collected A) using the custom-made cradle, B) using no cradle while the assessed hand remains still, and C) using no cradle and mimicking tremor.

The incorporation of the cradle in the spectra collection has been demonstrated to significantly enhance the precision of data acquisition, leading to reduced variability in the readings. Figure 5(A) illustrates that consecutive spectra obtained using the cradle yield three indistinguishable spectra with perfect overlap, indicating a high level of consistency. In contrast, Fig. 5(B), which depicts three assessments conducted without the use of a cradle on a stationary hand, and Fig. 5(C), simulating involuntary tremor movements without a cradle, both exhibit not only dissimilar spectral readings but also notable inter-spectral variation. This comparison underscores the critical role of the cradle in stabilizing the measurement process and ensuring the reliability of NIR spectroscopy data, particularly in the context of clinical and diagnostic applications where precision and replicability are paramount.

**Fig. 6.**
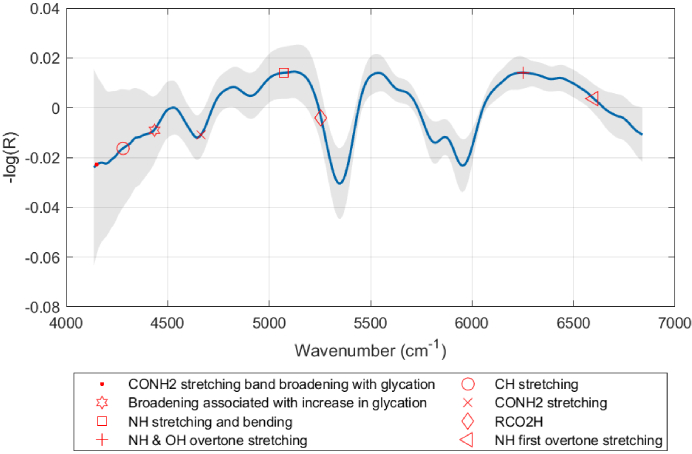
Mean NIR spectrum of pre-processed data (including standard normal variate (SNV), Asymmetric Least Square (AsLS) Correction and first derivative) and standard deviation (grey shade). Regions of interest (ROI) for glycation assessments have been highlighted. The ‘-log(R)’ axis reflects adjusted reflectance values due to preprocessing, extending beyond the typical 0 to 1 scale. This does not indicate reflectance >1 but results from not applying further normalization.

The Coefficient of Variation (CV) is a standardized measure of dispersion of a probability distribution or frequency distribution. It is defined as the ratio of the standard deviation (σ) to the mean (μ), expressed as a percentage. The CV allows for comparison of variability between datasets with different units of measure or different mean values, providing a contextually meaningful assessment of the relative variability. The mean spectrum and its associated standard deviation are depicted in Fig. 6. To ensure data consistency, the collected spectra per participant underwent thorough analysis, first for intra-hand coherence and subsequently for inter-hand comparison following outlier removal. The Coefficient of Variation (CV) was computed for all 60 spectra obtained from both the right and left hands of participants. Upon conducting a comparative analysis, it was observed that, irrespective of hand dominance, 92% of spectra exhibited negligible statistical differences in CV values between their hands. This finding underscores the robustness of the collected data, emphasizing its reliability in reflecting physiological similarities across participants’ hands.

Further scrutiny of CV across specific wavenumbers within the spectral data revealed minimal variation over 99% of the wavenumber range. This outcome further underscores the remarkable consistency and reliability of the spectral data, substantiating its utility in capturing and delineating physiological similarities between the hands of study participants.

The efficacy of handheld NIR devices for nail assessment is intricately linked to the transmission and reflection qualities, with considerations based on nail thickness. Nail plate thickness, typically around 0.5 mm in females and 0.6 mm in males, tends to increase with age, attributed to reduced growth rates and enlarged cell sizes. Cytological examinations of the dorsal nail plate have revealed a correlation between cell size, growth rate, and age [28].

Utilizing a handheld near-infrared (NIR) spectroscopy device for in vivo nail assessment proved to be a pragmatic methodology for exploring the biochemistry of nail tissue, considering the variable nail thickness. The inclusion of a diverse population in this study enabled us to validate that, even among very elderly adults with naturally thicker nails, the chosen methodology facilitated a successful and comprehensive NIR assessment. The utilization of this technique proves to be particularly advantageous as it can penetrate deeper layers (≥0.12 mm), a critical feature for exploring glycating products in the nail tissue, especially given that glycated products are significantly more prevalent in the deep layer of fingernails in close contact with blood vessels and interstitial fluid [29].

Varied pre-processing strategies, as detailed in Table 3, were systematically explored and compared. This exploration aimed to pinpoint and exploit the optimal ROI associated with glycated products, thereby facilitating the calibration of the Partial Least Squares Discriminant Analysis (PLS-DA) model developed for predictive purposes.

**Table 3.** Pre-processing optimization options

Pre-processing Method	Justification
Standard Normal Variate (SNV)	Standardizes spectra, mitigates baseline shifts, and enhances comparability among spectra.
Asymmetric Least Square (AsLS) Correction	Corrects baseline imperfections, ensuring a more accurate representation of spectral features.
Linear baseline correction	Implements linear baseline correction to rectify any remaining baseline distortions, refining the data for subsequent analysis.
Multiplicative Scatter Correction (MSC)	Compensates for scattering effects in the spectra, improving accuracy in the presence of physical variations.
1st Derivative	First derivative with quadratic enhancement enhances spectral features, aiding in identifying subtle changes in the dataset.
2nd Derivative	Second derivative amplifies fine spectral details, aiding in the discrimination of closely spaced peaks.

Nail plates are composed of three histological layers, each primarily made up of α-keratin [15]. This material features microfibrils of keratin proteins, embedded within a matrix of globular keratin-associated proteins (KAPs) [24]. Nail plates are predominantly composed of hard α-keratins, accounting for 80–90% of their structure, with the remainder consisting of soft α-keratins [24]. Hard keratins feature a more structured arrangement of microfibrils, giving the tissue its toughness. Conversely, soft keratins have cytoplasmic microfibrils arranged more loosely, lending mechanical resilience to epithelial cells [24]. The keratins in nails are distinct from those in the stratum corneum of the epidermis, particularly in their expression patterns and the absence of high-sulfur matrix components [28]. The presence of proteins rich in lysine residues on the nail makes them prone to glycation, becoming susceptible to react with amino groups of circulating serum proteins within the nail bed and the carbonyl group of glucose, producing fructosamine [29].

Given the majority composition of keratin in the structural composition of nails, interpreting the spectral contributions while not distinguishing among individual keratin variants leverages the collective insight that glycation affects keratins in a manner that is manifest in the observed spectral shifts. SDS-PAGE analysis, have demonstrated the non-glycated and glycated fractions of keratin within the nail and eye lens [29]. Keratins such as K1, K5, K6, K10, K14, K16, and K17, which are present in diverse segments of the nail, are characterized by a high propensity for lysine residues, rendering them particularly vulnerable to glycation [28]. This evidence collectively reinforces the discussion by highlighting how glycation distinctly influences the keratinous composition of nails, as evidenced through both spectral analysis and molecular weight differentiation. These signs of glycation [26] can be detected in the ROI shown in Fig. 6. Stages 1 and 2 of the data analysis elucidated the paramount importance of the ROI, highlighting their critical role in establishing meaningful associations with glycated proteins within the nail.

In the initial stages (stage 1 and stage 2), the analysis revealed that the top three performing models displayed commendable specificities, ranging from 83.1% to 98.5%. This indicates a robust capability to accurately identify negative cases. However, it should be noted that the sensitivities observed were relatively low, (2.9% - 47.4%), as detailed in Table 4 and Table 5. These findings underscore a potential area for enhancement in future model development, pointing towards an opportunity for refining these models to improve their detection of positive cases.

**Table 4.** ANODE01 chemometric model performance

Model	Sensitivity	Specificity	PPV	NPV	Concordance
	(95% CI) (DM, non-DM)	(95% CI) (DM, non-DM)	(95% CI) (DM, non-DM)	(95% CI) (DM, non-DM)	
M1 (N = 100)	47.4% (31.5%, 63.2%)	93.5% (87.4%, 99.7%)	81.8% (65.7%, 97.9%)	74.4% (64.7%, 84.0%)	76.00%
M2 (N = 100)	44.7% (28.9%, 60.5%)	91.9% (85.2%, 98.7%)	77.3% (59.8%, 94.8%)	73.1% (63.2%, 82.9%)	74.00%
M3 (N = 100)	39.5% (23.9%, 55.0%)	95.2% (89.8%, 100%)	83.3% (66.1%, 100%)	72.0% (62.2%, 81.7%)	74.00%

**Table 5.** ANODE02 chemometric model performance

Model	Sensitivity	Specificity	PPV	NPV	Concordance
	(95% CI) (DM, non-DM)	(95% CI) (DM, non-DM)	(95% CI) (DM, non-DM)	(95% CI) (DM, non-DM)	
M4 (N = 100)	25.7% (11.2%, 40.2%)	83.1% (74.0%, 92.2%)	45.0% (23.2%, 66.8%)	67.5% (57.2%, 77.8%)	63.00%
M5 (N = 100)	2.9% (0.0%, 8.4%)	98.5% (95.5%, 100%)	50.0% (0.0%, 100%)	65.3% (55.9%, 74.7%)	65.00%
M6 (N = 100)	20.0% (6.7%, 33.3%)	90.8% (83.7%, 97.8%)	53.8% (26.7%, 80.9%)	67.8% (58.0%, 77.6%)	66.00%

In our analysis, the skewness value of the data set was found to be 2.56, indicating a distribution with a pronounced tail towards the higher values despite the removal of outliers prior to training. This positive skewness may have contributed to a reduced sensitivity in our model, as it suggests a residual propensity for extreme values that could affect the threshold for positive detection. Nevertheless, our model demonstrated a robust performance in specificity, with results exceeding 90%. This high level of specificity suggests that the pre-processing step of outlier removal was effective in maintaining model integrity for true negative identification.

The analysis of the pooled data from both pilot studies (Table 6) highlights a significant trade-off between sensitivity and specificity across the models. While M7 and M9 have moderate sensitivity, all models exhibit high specificity, particularly M8. This implies that while they are less effective in identifying all true DM cases, they are highly efficient in ruling out non-DM cases. In the context of DM screening, this high specificity and NPV are particularly advantageous, as they reduce the likelihood of false positives, which can be a major concern in large-scale screening programs.

**Table 6.** ANODE 01 AND 02 pooled chemometric model performance

Model	Sensitivity	Specificity	PPV	NPV	Concordance
	(95% CI) (DM, non-DM)	(95% CI) (DM, non-DM)	(95% CI) (DM, non-DM)	(95% CI) (DM, non-DM)	
M7 (N = 200)	39.7% (28.5%, 51.0%)	90.6% (85.5%, 95.6%)	70.7% (56.8%, 84.7%)	72.3% (65.4%, 79.3%)	72.00%
M8 (N = 200)	34.2% (23.4%, 45.1%)	92.9% (88.5%, 97.4%)	73.5% (58.7%, 88.4%)	71.1% (64.2%, 78.0%)	71.50%
M9 (N = 200)	34.2% (23.4%, 45.1%)	90.6% (85.5%, 95.6%)	67.6% (52.5%, 82.7%)	70.6% (63.6%, 77.5%)	70.00%

Positive Predictive Value (PPV) refers to the proportion of individuals who test positive for diabetes through a screening test and are indeed diabetic. It is a crucial indicator of a test's accuracy in identifying true cases of diabetes among all positive diagnoses. Mathematically, PPV is expressed as the ratio of true positives to the sum of true positives and false positives. Conversely, Negative Predictive Value (NPV) denotes the proportion of individuals who receive a negative result from the diabetes screening test and do not have the disease. It measures the test's ability to correctly identify non-diabetic individuals among all those who test negative. NPV is calculated as the ratio of true negatives to the sum of true negatives and false negatives.

In our study, the application of PLS-DA was warranted despite the non-normal distribution of the data set. PLS-DA is known for its robustness in chemometric analyses and its ability to handle datasets with complex structures and a high number of predictors. The model’s emphasis on maximizing covariance between the predictors and the response variable allows for the accommodation of various data distributions, making it particularly suitable for the objectives of classification and prediction inherent in our study.

The parameterization of the PLS-DA was carefully considered in light of the asymmetric distribution observed, leveraging the flexibility of the PLS-DA method to maintain efficacy in the presence of such asymmetry. While this asymmetry poses interpretative challenges and raises the possibility of model overfitting, the adaptability of PLS-DA parameters played a pivotal role in preserving the robustness of our predictive analysis.

For subsequent research, it is imperative to explore data pre-processing strategies that can address skewness and non-normal distributions. The implementation of normalization transformations such as logarithmic, square root, or Box-Cox transformations could prove beneficial in reducing data asymmetry and enhancing the PLS-DA model’s performance. Employing statistical techniques that are resilient to deviations from normality may further refine the model's accuracy.

Moreover, future studies should prioritize securing borderline cases across the established HbA1c threshold. Ensuring a more precise classification of these borderline instances would aid in creating a more definitive separation between classes, which is crucial for the PLS-DA model's ability to fit and generalize effectively. This enhanced fitting would not only bolster the model's sensitivity and specificity but also strengthen its predictive validity for clinical applications. Such improvements are anticipated to advance the overall utility of the chemometric model, potentially leading to more accurate diagnostic tools in the diabetes field.

In the pursuit of enhancing the sensitivity without compromising our models’ specificity, we have identified several potential strategies for further optimization. The implementation of regularization techniques such as ridge regression (L2 regularization) and LASSO (L1 regularization) within the PLS-DA framework is anticipated to mitigate overfitting and enhance the model's generalization capabilities. This approach aims to reduce the influence of less pertinent predictors, thereby sharpening the focus on variables that significantly differentiate between the designated HbA1c thresholds. To address the challenge posed by dataset imbalance—predominantly skewed towards negative (<6.5% / 48 mmol/mol HbA1c) instances—we propose employing sampling techniques like the Synthetic Minority Over-sampling Technique (SMOTE) or adjusting class weights. These methods are expected to balance the dataset, consequently improving model sensitivity by enabling more accurate identification of true positives. The optimization of hyperparameters, including the learning rate through grid search and random search methods, represents a critical strategy for fine-tuning the model’s sensitivity. Exploring ensemble methods such as bagging and boosting by combining multiple PLS-DA models could leverage the strengths of individual models to achieve superior performance in detecting diabetic cases. Finally, external validation using datasets not previously encountered during the training phase will be crucial for assessing the model’s generalizability and pinpointing areas for sensitivity enhancement. These strategies collectively hold the promise of significantly improving the model's efficacy in identifying individuals at risk of diabetes, thereby contributing to more effective screening and early detection efforts.

The models’ high specificity and Negative Predictive Value (NPV) play a crucial role in mitigating patient anxiety, stigma, reducing unnecessary testing and over-diagnosis. Negative screening results have shown that it does not lead to complacency regarding health behaviors or perceptions of risk [30] while also preventing the stress and further invasive procedures that false positives can provoke. This approach not only fosters a patient-centric healthcare experience but also enhances trust in medical screenings. In addition, high specificity is crucial across various levels: it optimizes resource use in high-prevalence populations, is key in pre-employment and insurance examinations to mitigate negative impacts, and prevents over-medication and unnecessary interventions in the elderly. However, no method is perfect and as we use diagnostic tools for screening, if and where implemented, in the absence of appropriate channeling of only those at risk for diagnostic testing, even the standardized invasive and non-invasive methods currently used as the standard of care for the diagnosis of diabetes have a variable specificity for screening as ruling out those with no further need for diagnostic, exploratory testing. Due to the estimated high prevalence of underdiagnosed diabetes especially among people with limited access to public healthcare infrastructure and socioeconomic inequity, more affordable, accessible, and sustainable screening methods must be developed to curb the diabetes epidemic among these communities.

The precise exclusion of non-DM individuals allows for a more efficient allocation of limited healthcare resources, streamlining the diagnostic process and enhancing the cost-effectiveness of healthcare services. This accuracy also enables healthcare providers to focus on preventive measures for those without DM, guiding them towards healthier lifestyles to avert type 2 diabetes. Such a strategy benefits individual health and aligns with broader public health objectives, promoting equity and reducing diabetes incidence across various communities. However, it is recommended that efforts to improve the sensitivity of these models continue, to further enhance their efficacy in clinical applications.

The HbA1c test, while offering several benefits over other methods, may have limitations in sensitivity, cost, and availability, particularly in developing regions [31]. In response to these limitations, there is a growing interest in the development of reusable technologies that are not reliant on reagents, plastic disposable tubes and cartridges, have less stringent storage requirements and eliminate the need for highly specialized personnel, such as phlebotomists. Such technologies aim to offer accurate classification for diabetes screening, thus presenting a non-invasive and efficient alternative. This innovation has the potential to revolutionize current practices in diabetes diagnosis and monitoring. Crucially, it could significantly benefit populations experiencing health disparities and those with limited access to costly healthcare services. By addressing the specific challenges of these communities, the new tool could play a pivotal role in enhancing global diabetes care.

Screening for diabetes requires the use of a reliable method calibrated to detect levels of glycaemia without significant inter- or intra-individual variability in diverse populations with manifestations of concomitant conditions impacting the accuracy of methodology. There are also regional differences in the aptitude of healthcare systems to identify patients early but also, the use of non-standardized methodology for HbA1c detection.

An important factor enhancing the broader accessibility of technologies such as nail spectroscopy in increasingly diverse cohorts is the significantly lower melanocyte density in the nail apparatus compared to the skin. Melanocyte counts in the nail matrix epithelium range from 4 to 20 per mm^2^, in stark contrast to the 500 to 4500 melanocytes per mm^2^ typically found in normal glabrous skin [28]. This lower melanocyte density in nails suggests that nail analyses may be less influenced by variations in pigmentation. This distinctive feature positions nail spectroscopy as a potentially more inclusive and universally applicable technique in a variety of medical and scientific research contexts. The proposed system, which includes a Near-Infrared (NIR) spectrometer, a specialized cradle, and a robust chemometric model, demonstrates significant potential for incorporation into a comprehensive screening framework. This integration has potential to improve the efficiency and effectiveness of diabetes exclusion processes in primary care settings.

## 4. Limitations of the study

Clinically, one of the challenges in monitoring glycemia through glycated biomarkers such as nail keratin or other nail proteins is the potential interference from various nail conditions and contaminants. In addition, a higher incidence of nail affectation and nail conditions have been associated with long-term diabetes [32,33]. Therefore, the presence of nail abnormalities, discolorations, or any other visible changes in nail health renders the use of this technology for T2DM assessment largely impractical. This limitation underscores the need for clear nail health as a prerequisite for accurate but also, timely / early T2DM screening using the proposed nail spectroscopy method.

Nails exhibit a low water concentration, amounting to only a few percent, thereby establishing a nail matrix with a concentration ratio of interfering substances to protein that is negligible when compared to human serum or plasma [29]. However, the exposure of nail tissue to contaminants from cosmetic products to food, coupled with the potential mechanical damage and susceptibility to fungal infection, introduces additional challenges when evaluating the tissue quality and/or measurement preceding glycation assessments. The potential for these cases emphasizes the necessity of implementing a comprehensive quality control framework. This framework is essential for establishing a solid foundation for acquiring high-quality spectroscopic data, particularly in real clinical settings. Such a quality control system would involve stringent protocols for nail preparation, examination for contaminants or conditions that could skew results, and ensuring optimal conditions for spectroscopic analysis.

## 5. Conclusions

NIR Spectroscopy has proven to be a promising technique for evaluating glycated keratin in nails by leveraging subtle changes in molecular vibrations. The System under discussion, which integrates a NIR device and a specialized cradle, is designed to facilitate the deployment of NIR technology across an array of settings, including those staffed by healthcare personnel who may not have specialized training in spectroscopy. Consistency in delivery of the results is critical for building confidence and, by extension, the clinical decision-making processes based upon them. The reliability of physiological data captured by the device, underscores its potential as a dependable tool in physiological assessments.

The implementation of chemometric models into the system can facilitate the establishment of robust correlations between glycated keratin levels and clinical parameters, such as HbA1c values. While the proposed system and selected algorithm demonstrate a high degree of specificity and data reliability, its lower sensitivity raises important considerations for its application. The System's role in the detection of DM risk could be maximized in specific clinical scenarios where high specificity is prioritized, such as screening as the principle of successful, high-volume cost-effective, pragmatic and practical screening is to use a test/biomarker with high specificity in order to rule out those testing negative, i.e., reduce number of false positives and over-diagnosis.

This system presents a novel, non-invasive, and cost-effective approach for T2DM screening. By harnessing the synergy between advanced spectroscopic techniques and clinical parameters, it offers a comprehensive and personalized strategy for diabetes risk evaluation. The system's potential to enhance diabetes exclusion in primary care and broader healthcare initiatives is substantial, underscoring its value in contemporary diabetes management and prevention efforts.

## Data Availability

Data underlying the results presented in this paper are not publicly available at this time but may be obtained from the authors upon reasonable request.
